# Virtual Physician-Integrated Practice Units Enhance Pain Relief, Function, and Well-Being in Older Adults with Musculoskeletal Disorders: A Single-Arm Pre–Post Study

**DOI:** 10.3390/jcm15103675

**Published:** 2026-05-10

**Authors:** Elizabeth Peña, Linda Su, Mary I. O’Connor, Ryan A. Grant

**Affiliations:** Clinical Department, Vori Health, Inc., Nashville, TN 37204, USA; linda.su@vorihealth.com (L.S.); mary.oconnor@vorihealth.com (M.I.O.); ryan.grant@vorihealth.com (R.A.G.)

**Keywords:** chronic pain, geriatric, integrated practice unit, musculoskeletal pain, patient care team, physical therapy, rehabilitation, telemedicine, telerehabilitation

## Abstract

**Background/Objectives:** Age-related musculoskeletal (MSK) disorders lead to pain, reduced function, and diminished quality of life. This study aimed to evaluate the impact of a virtually delivered MSK care program on pain and function in older adults. **Methods**: A single-arm pre–post study was conducted analyzing self-reported outcomes of 902 patients aged ≥ 65 years who completed an initial evaluation with both an MSK specialty physician/nurse practitioner and a physical therapist, followed by two or more clinical visits. Outcome measures included pain (NPRS), function (SANE and PROMIS-10 GPH), and mental health (GAD-2, PHQ-2, and PROMIS-10 GMH). **Results**: Across 891 patients, mean pain intensity decreased from 4.56 ± 0.07 to 2.30 ± 0.07 (49.6%, *p* < 0.001) with 693 patients (77.8%) experiencing pain relief (63.2%, *p* < 0.001). In 172 patients with severe baseline pain (NPRS ≥ 7), 91.3% reported decreased pain (60.9%, *p* < 0.001). Functional gains were clinically meaningful, with SANE scores increasing from 53.85 ± 0.90 to 76.62 ± 0.75 (*n* = 795, 42.3%, *p* < 0.001). Pain reduction correlated with functional improvement (ρ = −0.29, *p* < 0.001) with modest correlations between engagement and improved pain (ρ = −0.09) and function (ρ = 0.23). Mental health outcomes remained stable, with marked improvement among patients with baseline symptoms of anxiety or depression, 44.2% and 57.1%, respectively. **Conclusions**: The virtual MSK care program produced meaningful improvements in pain and function while maintaining overall health. This highlights the potential for virtual MSK-focused integrated practice units (IPUs) to support holistic well-being and healthy aging in older adults.

## 1. Introduction

Musculoskeletal (MSK) disease is prevalent among older adults and can lead to pain, functional impairment, disability, and reduced quality of life, thus driving substantial healthcare utilization and economic burden [1]. Age-related musculoskeletal disorders such as osteoarthritis, spine degeneration, osteoporosis, and degenerative tendinopathies not only contribute to pain and limited function but also restrict participation in daily activities, diminish independence, increase fall risk, and adversely impact psychosocial health [2]. As the global population ages, the prevalence and societal impact of musculoskeletal conditions are expected to rise [3]. This highlights the urgent need for patient care models that are both effective and accessible for older adults. Traditional in-person rehabilitation and physical therapy are evidence-based, yet many older adults face significant barriers to accessing these services [4,5,6]. Transportation challenges, mobility limitations, geographic distance, and comorbidities can delay or prevent consistent engagement in therapy [7]. Integrated practice units (IPUs) represent a promising framework to address these challenges by organizing care and meeting patient needs in a more whole-person approach [8,9]. IPU-driven, multidisciplinary programs that incorporate physicians, physical therapists, health coaches, and registered dieticians unify medical and physical therapy plans of care with behavioral change and lifestyle support and have the potential to transform outcomes for older adults with musculoskeletal conditions [10,11,12]. Such integrated care models have been associated with improved outcomes, enhanced patient engagement, and more personalized care [9].

Most prior studies have focused primarily on musculoskeletal outcomes, without systematically evaluating associated mental health or broader physical health measures, despite the well-recognized interplay between chronic pain, anxiety, depression, and functional capacity. Moreover, although telehealth-delivered rehabilitation has shown promise in general adult populations, few studies have specifically examined its effectiveness in adults aged 65 years and older—a population that faces unique barriers to in-person care and may derive particular benefit from virtual delivery models. This gap is notable given that older adults represent the fastest-growing segment of patients with chronic MSK conditions and are disproportionately affected by access-to-care barriers. In this study, we evaluated a physician-integrated MSK care program for older adults within a coordinated telehealth platform. The program combined medical oversight, functional assessment, individualized physical therapy, cognitive behavioral wellness coaching, and nutritional counseling. Our objectives were to quantify changes in pain intensity (Numeric Pain Rating Scale [NPRS]), functional status (Single Assessment Numeric Evaluation [SANE] complemented by Patient-Reported Outcomes Measurement Information System-10 [PROMIS-10] Global Physical Health [GPH]), and mental health outcomes (Generalized Anxiety Disorder-2 [GAD-2], Patient Health Questionnaire-2 [PHQ-2], and PROMIS-10 Global Mental Health [GMH]), while exploring the influence of patient engagement and clinical visit patterns on outcomes. By examining outcomes across pain, function, and health-related quality of life, this study highlights the potential for virtual IPU frameworks to deliver high-value, patient-centered MSK care that improves patient prognosis, promotes sustained engagement, and enhances overall well-being for older adults with complex needs.

## 2. Materials and Methods

### 2.1. Study Population and Selection Criteria

We conducted a single-arm pre–post study using retrospectively collected data from adult patients aged 65 years and older with MSK symptoms suitable for virtual care and access to the necessary technology needed to complete synchronous audio–video virtual telehealth visits. Patients completed an initial evaluation between 1 September 2023 and 10 February 2025, with a minimum of two follow-up clinical visits extending through 30 June 2025. The initial evaluation involved a combined assessment by both a specialty MSK medical provider (physician or nurse practitioner) and a physical therapist within the same virtual session. Patients who completed their initial baseline evaluation with only one clinical provider, either a physician, nurse practitioner, or physical therapist, were excluded from this study. Patients were required to complete at least two additional follow-up visits, conducted as one-on-one sessions for individualized physical therapy, health coaching, medical nutrition therapy, or medical follow-up.

Integrated care coordination was embedded throughout the treatment pathway. Patients with a positive mental health screen (GAD-2 and/or PHQ-2) were referred for cognitive behavioral interventions delivered by health coaches, who supported overall well-being through mindfulness, sleep hygiene, goal setting, stress management, and lifestyle optimization. Registered dietitians addressed nutritional needs and healthy weight management. When clinically indicated, patients were referred for diagnostic imaging, surgical consultation, or licensed mental health services. Patients who subsequently underwent surgical or procedural intervention(s) remained included in the analysis.

**Figure 1:**
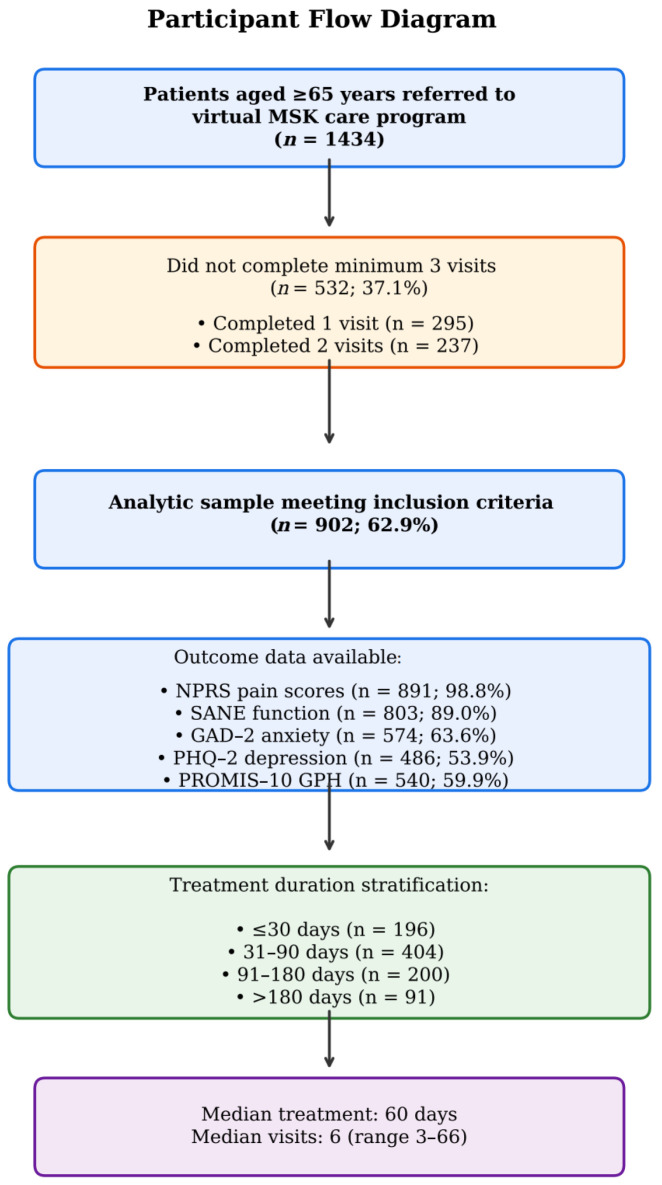
Participant flow diagram illustrating the progression for patients who initiated the program through exclusion criteria to the final analytic sample.

Exclusion criteria included patients who lacked the required combined (medical provider plus physical therapist) baseline assessment visit, completed fewer than two follow-up visits, presented with an MSK condition not suitable for virtual physical therapy, or developed a second MSK condition during their course of treatment. Of 1434 unique patients aged ≥ 65 years who initiated the program, 532 (37.1%) did not complete the minimum of three total visits required for inclusion (295 completed one visit, 237 completed two visits). A total of 902 patients (62.9%) met all inclusion criteria and constituted the analytic sample (Figure 1). The median treatment duration for included patients was 60 days (mean 87.5 ± 84.7 days; range 6–623 days), with a median of 6 total completed clinical visits (mean 8.3 ± 6.6; range 3–66). When stratified by treatment duration, NPRS improvement showed a significant dose–response pattern (Kruskal–Wallis H = 19.17, *p* < 0.001): patients treated for ≤30 days showed a mean NPRS reduction of 1.71 ± 2.27 points (*n* = 196), those treated for 31–90 days improved by 2.29 ± 2.32 points (*n* = 404), those treated for 91–180 days improved by 2.63 ± 2.42 points (*n* = 200), and those treated for more than 180 days improved by 2.58 ± 2.38 points (*n* = 91). SANE functional improvement followed a similar dose–response pattern (Kruskal–Wallis H = 56.00, *p* < 0.001), with mean improvements of 14.2, 23.4, 29.1, and 29.5 points across the same duration categories, respectively.

### 2.2. Assessment Measures and Outcome Definitions

Primary outcomes were pain intensity, measured via the NPRS, and functional status, assessed using SANE. NPRS is a patient-reported outcome measure widely used to quantify subjective pain intensity. It consists of an 11-point whole-number scale ranging from 0 (no pain) to 10 (worst pain ever) [13,14,15]. The SANE score is a widely adopted, patient-reported outcome measure that provides a concise and sensitive assessment of perceived functional status, particularly in musculoskeletal and orthopedic populations. SANE scores represent the perceived function of an affected body part as a percentage of normal on a 0–100 scale, where 0 indicates complete loss of function and 100 indicates normal function [16,17,18]. Secondary outcomes included GAD-2, PHQ-2, and PROMIS-10. GAD-2 and PHQ-2 are validated mental health screening tools with scores ≥3 indicating clinically important thresholds for further assessment and appropriate mental health referrals [19]. PROMIS-10 is a standardized, validated 10-item questionnaire that assesses both global physical health and global mental health from the patient’s perspective [20,21]. Each item on the questionnaire is rated on a 5-point scale and converted into standardized T-scores (mean = 50 and SD = 10) to allow for comparison to the general United States (US) population with higher scores indicating better health [20]. The PROMIS-10 GPH score offers a validated, standardized measure of general physical health, providing broader context for functional recovery beyond the affected musculoskeletal region by assessing overall physical health, physical function, pain, and fatigue [20]. Table 1 summarizes the patient-reported outcome measures included in the study.

Data were excluded if baseline or post-intervention outcome measures were missing or if assessments were completed less than three days apart. These criteria ensured inclusion of participants with interpretable outcomes reflective of the virtual MSK care intervention. Missing data rates varied across outcome measures; complete paired data were available for 891 of 902 patients (98.8%) for NPRS, 803 (89.0%) for SANE, 574 (63.6%) for GAD-2, 486 (53.9%) for PHQ-2, and 540 (59.9%) for PROMIS-10 GPH. Analyses were conducted using available paired data for each measure (complete-case analysis). No imputation was applied for missing values, as missingness was assumed to result primarily from variations in survey administration timing rather than outcome-related factors. No formal a priori sample size calculation was performed; this study was designed as an exploratory analysis of all eligible patients within the specified enrollment period. Post hoc power analysis indicated that the achieved sample sizes provided greater than 99% power to detect the observed effect sizes for NPRS (Cohen’s d = 0.96, *n* = 891) and SANE (d = 0.93, *n* = 751) at α = 0.05. For secondary outcomes, power was 100% for PROMIS-10 GPH (d = 0.47, *n* = 540), 85% for PHQ-2 (d = 0.14, *n* = 486), and 48.4% for GAD-2 (d = 0.08, *n* = 574), indicating that the study was adequately powered to detect clinically meaningful changes in primary outcomes but had limited power to detect small effects in GAD-2 scores. We addressed missing data using multiple imputation by chained equations (m = 10 imputations). The iterative model used age, total completed visits, and baseline and follow-up outcomes as covariates; final estimates were pooled according to Rubin’s rules. Additionally, a conservative lower bound of the treatment effect was calculated via a sensitivity analysis that imputed “no change” for all missing follow-up data.

### 2.3. Statistical Analysis

This single-arm pre–post study employed quantitative methods to evaluate changes in clinical outcomes and examine associations among health-related variables. All statistical analyses were performed using IBM SPSS Statistics, Version 29 (IBM Corp., Armonk, NY, USA). Descriptive statistics were calculated for baseline demographic and clinical characteristics.

Within-subject changes from baseline to post-intervention were assessed using two-tailed paired *t*-tests, with statistical significance set at *p* < 0.05. All results include standard error of the mean, whereas age and clinical visit length data are presented as mean and SD. Analyses included only complete paired data with both pre- and post-intervention measurements. Normality of continuous variables was assessed using the Kolmogorov–Smirnov test for samples >50 and the Shapiro–Wilk test for smaller samples. When normality assumptions were violated (*p* < 0.05), non-parametric statistical methods were employed to ensure analytical validity.

Associations among continuous and ordinal variables, including pain intensity, functional status, and patient-reported outcomes, were evaluated using Spearman’s rank-order correlation coefficients. This non-parametric approach was selected to accommodate the ordinal nature of certain outcome measures and potential non-normal distributions characteristic of clinical data.

Comparisons across multiple anatomical regions or clinical subgroups were conducted using Kruskal–Wallis tests, a non-parametric alternative to one-way analysis of variance appropriate for ordinal outcomes and non-normal distributions. When omnibus tests achieved statistical significance, post hoc pairwise comparisons were performed with Bonferroni correction to control for multiple testing and minimize Type I error.

## 3. Results

### 3.1. Participant Demographics

The study cohort included 902 participants, with a mean age of 73.6 ± 6.1 years and a range of 65–97 years (Table 2). Gender distribution was 50.5% female (*n* = 456), 48.7% male (*n* = 439), and 0.8% other/undisclosed (*n* = 7). Racial data was limited, as more than half of participants (68.1%, *n* = 614) either did not disclose their race or did not respond. Among those participants who provided information, White participants formed the largest subgroup (30.6% of the total sample, *n* = 276), followed by Black or African American participants (0.9%, *n* = 8) and Asian participants (0.4%, *n* = 4).

### 3.2. Clinical Presentations

Back pain was the most common presenting complaint, affecting 43.9% of 902 participants (Table 3). Other frequent conditions included knee or lower leg pain (14.0%, *n* = 126), shoulder or upper arm pain (12.4%, *n* = 112), neck pain (9.6%, *n* = 87), and pelvis/hip/thigh pain (9.0%, *n* = 81). Less common presentations involved wrist/hand/finger pain (4.4%, *n* = 40), ankle/foot/toe pain (4.1%, *n* = 37), elbow pain (1.0%, *n* = 9), and whole-body or other musculoskeletal pain (1.6%, *n* = 14). Most participants (77.3%, *n* = 697) reported chronic symptoms (≥3 months), while 14.6% (*n* = 132) presented with acute/subacute symptoms, and 2.0% (*n* = 18) experienced recurrent symptoms. Symptom duration was unspecified or could not be classified in 6.1% of participants (*n* = 55).

### 3.3. Treatment Engagement

Participants completed a mean of 8.3 ± 6.6 visits (median = 6; range: 3–66). This wide variation in visit frequency reflected differences in symptom acuity, patient self-efficacy, diagnostic complexity, and baseline functional impairment. Interventions were individualized to meet patient-specific needs; therefore, participants with more severe impairments often required additional one-on-one sessions to achieve meaningful recovery.

### 3.4. Pain Outcomes

Across the full pain subcohort of 891 patients, mean pain scores decreased significantly from 4.56 ± 0.07 at baseline to 2.30 ± 0.07 post-intervention (*p* < 0.001), representing a 49.6% overall improvement. In the subgroup of 597 patients who presented with moderate to severe baseline pain (NPRS ≥ 4), mean pain scores decreased from 5.73 ± 0.06 to 2.71 ± 0.09 (*p* < 0.001), a 52.8% reduction. Among the 172 patients with severe baseline pain (NPRS ≥ 7), mean pain scores dropped from 7.71 ± 0.06 to 3.47 ± 0.21(*p* < 0.001), a 55.0% overall reduction in pain intensity.

**Figure 2:**
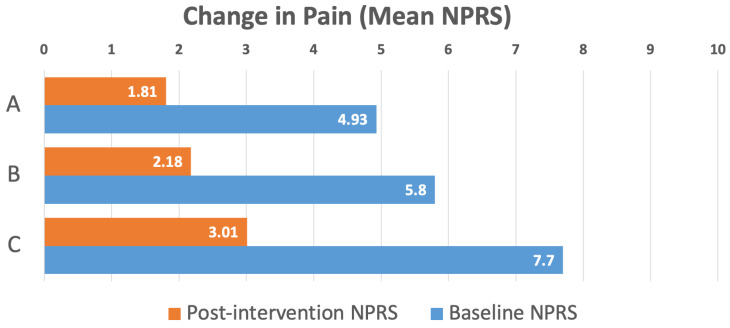
Change in pain (mean NPRS scores) from baseline to post-intervention for patients whose NPRS scores improved. (A) Full pain subcohort, *n* = 693 (out of 891 patients), 63.2% reduction, *p* < 0.001, (B) moderate to severe baseline pain subgroup, *n* = 514 (out of 597 patients), 62.4% reduction, *p* < 0.001, and (C) severe baseline pain subgroup, *n* = 157 (out of 172 patients), 60.9% reduction, *p* < 0.001.

Among the pain subcohort, 693 of the total 891 patients (76.8%) experienced an improvement in their NPRS pain score, with mean scores among this subgroup declining from 4.93 ± 0.07 to 1.81 ± 0.06 (*p* < 0.001), a 63.2% reduction, underscoring the intervention’s broad efficacy across varying baseline pain severities. Among the 597 patients with moderate to severe baseline pain, 514 patients (86.1%) demonstrated a reduction in their NPRS pain score, with mean scores among this subgroup of 514 patients declining from 5.80 ± 0.06 to 2.18 ± 0.08 (*p* < 0.001), a 62.4% reduction in pain, demonstrating consistent effectiveness in managing moderate to severe pain. Notably, 157 of the 172 patients (91.3%) with severe baseline pain experienced pain relief, with mean pain scores among this group of 157 patients decreasing from 7.70 ± 0.06 to 3.01 ± 0.19 (*p* < 0.001), reflecting a 60.9% per-patient improvement (Figure 2). These results highlight the program’s transformative effect on severe pain in the geriatric population.

Spearman rank-order correlation analysis revealed a modest but statistically significant negative association between total clinical visits and pain scores (ρ = −0.09, *p* = 0.009), suggesting that greater engagement in care was linked with larger reductions in pain. Improvements in pain were significantly correlated with functional gains as measured by the SANE score (ρ = −0.30, *p* < 0.001), emphasizing the intervention’s dual impact on both pain intensity and functional recovery.

Analysis of pain reduction by anatomical region using the Kruskal–Wallis test yielded a near-significant trend (*p* = 0.054), with exploratory pairwise comparisons indicating potential differences that did not persist after Bonferroni correction. These observations may reflect limited power in smaller regional subgroups and highlight the need for larger studies to clarify potential regional variability in pain outcomes.

Overall, the intervention produced substantial, clinically meaningful pain reduction across all patient subgroups with particularly pronounced effects in those with severe baseline pain. The findings suggest that sustained engagement in multidisciplinary care may be associated with favorable outcomes, though the single-arm design limits definitive conclusions about the role of structured, integrated interventions in restoring function and improving quality of life in older patients with significant musculoskeletal pain.

### 3.5. Functional Outcomes

**Figure 3:**
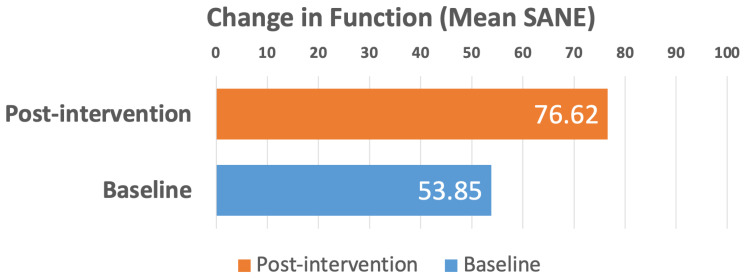
Change in function (mean SANE scores) from baseline to post-intervention for the 795 patient SANE responses that were analyzed, 42.3% improvement, *p* < 0.001.

A total of 795 patient SANE responses were analyzed, with scores spanning the full 0–100 range. Baseline SANE scores averaged 53.85 ± 0.90 and improved to 76.62 ± 0.75 post-intervention, representing robust and statistically significant functional gains (42.3%, *p* < 0.001) (Figure 3). Notably, 609 patients (76.6%) demonstrated improved SANE scores, highlighting the broad effectiveness of the intervention across a diverse cohort.

Functional status, as measured by SANE, was complemented by self-reported global physical health status using the PROMIS-10 GPH T-score in 540 patients. Mean scores increased modestly from 44.57 ± 0.31 at baseline to 46.94 ± 0.31 post-intervention (*p* < 0.001), indicating stable or slightly improved physical health alongside substantial functional recovery. This stability underscores the program’s holistic design, achieving meaningful functional improvements without compromising general physical well-being.

Analysis of engagement revealed a modest but statistically significant positive correlation between the number of clinical visits and functional improvement (Spearman ρ = 0.23, *p* < 0.001), suggesting that greater participation in care was associated with superior outcomes. Functional gains also aligned with reductions in pain, with a significant inverse correlation between SANE scores and pain scores (ρ = −0.29, *p* < 0.001), further demonstrating the intervention’s dual impact on both pain relief and functional status.

Regional analysis using a Kruskal–Wallis test found no significant differences in SANE improvements across anatomical regions (*p* = 0.24). Although some post hoc comparisons initially suggested regional differences, none remained significant after Bonferroni correction for multiple comparisons. These findings suggest the intervention’s effectiveness is consistent across musculoskeletal sites.

Collectively, these results demonstrate that the program delivers substantial and clinically meaningful improvements in patient-reported functional status while maintaining overall physical health. The mean NPRS reduction of 2.26 points exceeded the established minimal clinically important difference (MCID) threshold of 2.0 points for chronic pain, and the SANE improvement of 22.8 percentage points represents a clinically meaningful gain in perceived function. However, the clinical significance of changes in secondary outcomes warrants further investigation against established MCID thresholds. The findings suggest value in a holistic, patient-centered approach that integrates functional recovery, pain management, and sustained engagement to enhance health-related quality of life, particularly for older individuals with severe baseline limitations.

### 3.6. Mental Health Outcomes

A total of 574 patients with GAD-2 scores ranging from 0 to 6 were included in the analysis. The anxiety subcohort demonstrated minimal baseline anxiety, with a mean score of 0.85 ± 0.05. Despite the low starting point, program completion yielded a statistically significant reduction to 0.76 ± 0.05 (*p* < 0.001), confirming measurable improvement in anxiety symptoms across the population.

**Figure 4:**
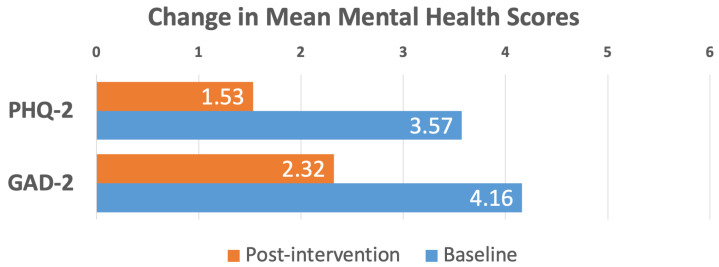
Change in mean mental health scores (PHQ-2 and GAD-2) from baseline to post-intervention for patients who presented with clinically relevant symptoms of depression (PHQ-2 ≥ 3, *n* = 30, 57.1% improvement, *p* < 0.001) and anxiety (GAD-2 ≥ 3, *n* = 44, 44.2% improvement, *p* < 0.001).

Among the 44 patients with clinically relevant anxiety symptoms at baseline (GAD-2 ≥ 3; range: 3–6), mean scores decreased from 4.16 ± 0.16 to 2.32 ± 0.27 post-intervention (*p* < 0.001), representing a 44.2% improvement and bringing mean anxiety levels below the clinically relevant screening threshold (Figure 4).

For depression, 486 patients with PHQ-2 scores (range: 0–6) were analyzed. Overall, the sub-cohort exhibited minimal depressive symptoms at baseline (0.69 ± 0.05), which remained stable at program completion (0.55 ± 0.04, *p* = 0.003), indicating maintenance of mental well-being without deterioration. Among the 30 patients with clinically significant baseline depressive symptoms (PHQ-2 ≥ 3), substantial improvement was observed with mean scores decreasing from 3.57 ± 0.13 to 1.53 ± 0.25 (*p* < 0.001, 57.1% reduction) (Figure 4). PROMIS-10 GMH T-scores similarly reflected stability, with baseline and completion scores of 50.91 ± 0.33 and 50.95 ± 0.34, respectively (*p* = 0.82). These findings highlight the program’s capacity to meaningfully alleviate moderate to severe anxiety and depressive symptoms while maintaining overall mental health in the broader cohort.

### 3.7. Sensitivity Analyses

Sensitivity analyses were performed to evaluate the robustness of our primary findings against selection bias (from non-completion of the three-visit minimum) and missing data in secondary outcomes. To assess selection bias, we compared baseline characteristics between the 902 included patients and the 532 patients (aged ≥65 years) who initiated care but completed fewer than three visits. There were no significant differences in mean age between the groups (73.6 ± 6.1 vs. 73.2 ± 5.7 years, *p* = 0.17). To quantify the impact of attrition, a “worst-case” intention-to-treat analysis was performed under the deliberately conservative and clinically implausible assumption that all 532 excluded patients experienced zero improvement. In other words, this analysis pretends that every patient who completed less than 3 visits received no benefit at all, regardless of how briefly they participated, and combines those assumed null outcomes with the observed outcomes of completers to produce a pooled estimate that represents a deliberate lower bound on the true treatment effect. Additionally, we know some patients only need to complete 1 or 2 visits and can then turn to self-management. Regardless, even under this extreme stress test, the pooled NPRS reduction was 1.42 points (*n* = 1423, *p* < 0.001) and SANE improvement was 13.5 points (*n* = 1283, *p* < 0.001), with both effects remaining highly statistically significant. For context, the 2.0-point minimal clinically important difference (MCID) for chronic pain represents the smallest change on the NPRS that patients perceive as a meaningful improvement in their everyday pain. The worst-case NPRS estimate of 1.42 falls just below this threshold precisely because it assumes a complete absence of benefit in over a third of the cohort; if excluded patients achieved as little as 1.55 points of improvement on average (approximately 68% of the 2.27-point reduction observed in completers), the pooled effect would reach MCID. A tipping-point analysis, which asks how badly the excluded patients would have had to fare for the overall conclusion to be overturned, indicated that the 532 excluded patients would need to have experienced a mean worsening of 3.80 NPRS points to nullify the pooled effect entirely, a degree of clinical deterioration with no precedent in this population. Together, these two analyses bracket the plausible range of the true treatment effect: a value below the worst-case lower bound is implausible because it would require zero benefit in every excluded patient, and a value that fails to reach MCID would require substantial deterioration in the excluded group rather than even the modest improvement expected from a brief care episode. The most reasonable interpretation is therefore that the true treatment effect lies at or above the MCID threshold. Finally, multiple imputation (m = 10) confirmed the primary findings (NPRS pooled difference 2.25 points, *p* < 0.001; PROMIS-10 GPH pooled difference −2.40 points, *p* < 0.001), but the small changes observed in GAD-2 (pooled difference 0.09 points, *p* = 0.082) and PHQ-2 (pooled difference 0.09 points, *p* = 0.066) lost statistical significance under the more rigorous handling of missing data. Both pooled *p*-values, however, remained below the *p* < 0.10 threshold, indicating a trend toward improvement rather than a null result; these mental health findings offer an exploratory perspective that remains sensitive to the specific data assumptions applied.

## 4. Discussion

This study demonstrates that a structured virtual MSK care program can deliver significant improvements in pain, functional status, and select mental health outcomes in adults aged 65 years and older. Importantly, the intervention yielded clinically meaningful pain reduction across the full pain subcohort, with pronounced effects in the subgroups of patients with moderate to severe baseline pain and severe baseline pain. This finding underscores the program’s potential to transform outcomes for older patients with intense pain experiences that may negatively impact their daily activities, functional mobility, and mental health.

Functional status, as measured by SANE, paralleled pain improvement, with a 42% average improvement in patient-reported functional capacity. PROMIS-10 GPH scores remained stable or improved slightly, indicating that targeted functional gains were achieved without compromising overall physical well-being. The observed correlations between clinical engagement, pain reduction, and functional improvement suggest that sustained participation in structured multidisciplinary care amplifies patient outcomes. Furthermore, the program demonstrated consistent efficacy across multiple anatomical regions, supporting its broad applicability.

Mental health outcomes remained stable in the overall population, reflecting maintenance of psychological well-being during the intervention. However, among those patients with clinically significant anxiety and/or depressive symptoms at baseline, substantial improvements were observed. This highlights the program’s capacity to support mental health through an integrated approach and emphasizes the importance of incorporating mindfulness and lifestyle interventions, particularly in older adults where comorbid psychosocial factors can exacerbate both pain and functional limitations.

Collectively, these results support a holistic, patient-centered model of care that simultaneously addresses pain, function, and emotional well-being. The integration of physicians, physical therapists, health coaches, and dietitians within a virtual platform appears to facilitate personalized, effective interventions that can be delivered at scale, overcoming barriers to traditional in-person care.

These findings compare favorably to published benchmarks for musculoskeletal rehabilitation. In the broader physical therapy literature, only 43% of patients complete their prescribed program [22], and 73% of patients with musculoskeletal conditions miss at least one appointment during their care episode [23]. By contrast, the present study demonstrated a 62.9% program completion rate among older adults aged ≥65, suggesting that the virtual interdisciplinary model may enhance treatment adherence in a population that typically faces significant barriers to in-person care access. Among the 532 patients who did not complete the minimum three visits, literature-based estimates suggest that approximately 13% of early discontinuers may have stopped because they felt sufficiently improved without formal discharge [22], while logistic and accessibility factors account for a substantial proportion of attrition. Notably, the present cohort achieved a mean pain reduction of 2.26 points on the NPRS (77.8% of patients improving), which compares favorably to the 64% improved outcome rate reported for Medicare beneficiaries with musculoskeletal conditions receiving an average of 6.8 visits [24]. The literature further indicates that approximately 71% of total functional improvement occurs in the first 45% of the treatment episode [25], and that early response within two to three visits is a strong predictor of eventual treatment success [26]. These patterns suggest that even among patients who completed only one or two visits before discontinuation, a proportion may have experienced early improvement consistent with appropriate treatment response. 

Several important limitations of this study should be considered. First, as a single-arm pre–post study without a control group, the observed improvements cannot be causally attributed to the intervention alone. Regression to the mean, natural disease progression, placebo effects, and other time-related confounding factors may have contributed to the observed changes. Future randomized controlled trials or matched-cohort studies are needed to establish comparative effectiveness. Second, the inclusion criteria requiring completion of at least three visits may have introduced selection bias by preferentially including more adherent and motivated patients. Of 1434 unique patients aged ≥ 65 who initiated care, 532 (37.1%) did not complete the required inclusion criteria for follow-up visits and were excluded, which may overestimate treatment effectiveness. Third, all outcomes are self-reported, which introduces potential recall and response biases. Fourth, missing data rates were substantial for secondary outcomes (ranging from 36% to 46% for GAD-2, PHQ-2, and PROMIS-10), and the complete-case analysis approach may introduce bias if data were not missing completely at random. However, sensitivity analyses using worst-case imputation (assuming no improvement for all patients with missing data) confirmed that primary findings remained robust: NPRS results remained highly significant (*t* = 28.58, *p* < 0.001) and SANE results likewise remained significant (*t* = 23.77, *p* < 0.001) under this conservative assumption. Fifth, all study participants were required to have access to an electronic device such as a computer, tablet, or phone, and a reliable Wi-Fi connection to complete virtual telehealth visits, possibly limiting generalization to the broader geriatric population. Lastly, the study does not assess the durability of outcomes beyond the intervention period; therefore, there is a lack of long-term outcome data needed to better understand maintenance of pain and functional gains over time.

While 37.1 percent of participants did not reach the three-visit threshold, rigorous sensitivity analyses including worst-case and tipping-point scenarios demonstrated that the primary pain and functional improvements remained statistically and clinically robust. Although the sample naturally reflects highly motivated individuals, these results provide a clear proof of concept for the effectiveness of virtual care among engaged older adults. Interestingly, several patients in the excluded group achieved the ability to self-manage after only one or two visits, presenting a compelling opportunity for future study. For the purposes of this analysis, the findings should be interpreted as primarily applicable to older adults with sufficient engagement to complete a multi-visit virtual care episode. Furthermore, while primary outcomes showed consistent strength under multiple imputation, the secondary mental health outcomes (GAD-2 and PHQ-2) only trended to significance after sensitivity analyses and should be viewed as exploratory and sensitive to missing-data assumptions.

The positive end-of-program results provide a strong foundation for future longitudinal research. Prospective follow-ups at 6, 12, and 24 months are essential next steps to verify the durability of treatment benefits and identify the specific factors driving sustained success. Future studies should reassess both primary and secondary outcome batteries to include NPRS, SANE, PROMIS 10, GAD 2, and PHQ 2 (or PHQ 9) while incorporating additional metrics such as symptom recurrence, healthcare utilization, and surgical intervention rates to fully capture the long-term clinical impact of the program.

To further elevate the quality of evidence, future research should employ randomized or matched control designs to establish comparative effectiveness and utilize intention to treat analyses with appropriate data imputation for missing values. Expanding access to more diverse patient populations will be critical for maximizing generalizability and clinical impact. Finally, all observed changes must be evaluated against established MCID thresholds to ensure that the statistical gains translate into meaningful improvements in patient care.

## 5. Conclusions

This study suggests that digital, multidisciplinary MSK care is associated with improvements in pain and function while maintaining or enhancing overall physical and mental health in older adults. The intervention was particularly effective for patients with severe baseline impairments, demonstrating the potential for meaningful improvement among those with greater baseline impairment. Sustained clinical engagement emerged as an important contributor to outcomes, highlighting the value of structured, patient-centered programs. These findings underscore the promise of virtual IPUs to restore function, reduce pain, and support holistic well-being in geriatric populations. However, these results should be interpreted in light of the single-arm study design, the potential for selection bias from excluding patients who did not complete at least three visits, and the reliance on self-reported outcome measures. Future randomized controlled trials are needed to confirm these findings and establish comparative effectiveness.

## Figures and Tables

**Table 1:** Summary of patient-reported outcome measures included in the study.

**Outcome Measure**	**Question**	**Interpretation**
Numeric Pain Rating Scale (NPRS)	How would you rate your average pain over the last 24 h from 0–10 with 0 being no pain and 10 being your worst pain ever?	Mild pain: 0–3 Moderate pain: 4–6 Severe pain: 7–10
Single Assessment Numeric Evaluation (SANE)	How would you rate your painful area as a percentage of normal from 0–100 with 100% being completely normal?	Higher scores reflect better perceived function.
Patient Health Questionnaire-2 (PHQ-2)	Over the last 2 weeks, how often have you been bothered by any of the following problems? 1. Little interest or pleasure in doing things 2. Feeling down, depressed, or hopeless	Score ≥3 is the threshold for a positive screen for clinically relevant depressive symptoms requiring further assessment.
Generalized Anxiety Disorder-2 (GAD-2)	Over the last 2 weeks, how often have you been bothered by the following problems? 1. Feeling nervous, anxious, or on edge 2. Not being able to stop or control worrying	Score ≥3 is the threshold for a positive screen for clinically relevant anxiety symptoms requiring further assessment.
Patient-Reported Outcomes Measurement Information System-10 (PROMIS-10)	1. In general, would you say your health is: 2. In general, would you say your quality of life is: ^1^ 3. In general, how would you rate your physical health? ^2^ 4. In general, how would you rate your mental health, including your mood and your ability to think? ^1^ 5. In general, how would you rate your satisfaction with your social activities and relationships? ^1^ 6. To what extent are you able to carry out your everyday physical activities such as walking, climbing stairs, carrying groceries, or moving a chair? ^2^ 7. How would you rate your pain on average? ^2^ 8. How would you rate your fatigue on average? ^2^ 9. In general, please rate how well you carry out your usual social activities and roles? (This includes activities at home, at work, and in your community, and responsibilities as a parent, child, spouse, employee, friend, etc.) 10. How often have you been bothered by emotional problems such as feeling anxious, depressed, or irritable? ^1^	PROMIS-10 produces two scores: Global Physical Health (GPH) and Global Mental Health (GMH). T-score of 50 represents the average physical and mental health of the US general population.

^1^ PROMIS-10 items 2, 4, 5, and 10 are used to calculate GMH T-score. ^2^ PROMIS-10 items 3, 6, 7, and 8 are used to calculate GPH T-score.

**Table 2:** Participant demographics (*N* = 902).

Age (Years)	
Range	65–97
Mean ± SD	73.6 ± 6.1
Sex, *n* (%)	
Female	456 (50.5)
Male	439 (48.7)
Other/undisclosed	7 (0.8)
Race, *n* (%)	
Asian	4 (0.4)
Black or African American	8 (0.9)
White	276 (30.6)
Did not disclose or no response	614 (68.1)

**Table 3:** Clinical presentations (*N* = 902).

Presenting Complaint, *n* (%)	
Back pain	396 (43.9)
Knee or lower leg pain	126 (14.0)
Shoulder or upper arm pain	112 (12.4)
Neck pain	87 (9.6)
Pelvis/hip/thigh pain	81 (9.0)
Wrist/hand/finger pain	40 (4.4)
Ankle/foot/toe pain	37 (4.1)
Elbow pain	9 (1.0)
Whole-body or other musculoskeletal pain	14 (1.6)
Symptom Duration, *n* (%)	
Chronic (≥3 months)	697 (77.3)
Acute/subacute (<3 months)	132 (14.6)
Recurrent	18 (2.0)
Unspecified	55 (6.1)

## Data Availability

Data is unavailable due to privacy restrictions.

## Abbreviations

The following abbreviations are used in this manuscript:

GAD-2	Generalized Anxiety Disorder-2
GMH	Global Mental Health
GPH	Global Physical Health
IPU	Integrated practice unit
MSK	Musculoskeletal
NPRS	Numeric Pain Rating Scale
PHQ-2	Patient Health Questionnaire-2
PROMIS-10	Patient-Reported Outcomes Measurement Information System-10
SANE	Single Assessment Numeric Evaluation
US	United States
